# Effect of decompression and online transmission on the dimensional changes of .stl files generated by intra-oral scanning

**DOI:** 10.1371/journal.pone.0272989

**Published:** 2022-09-15

**Authors:** Juliano Martins Bueno, Carolina Guarniéri Gouveia, Mayara Barbosa Viandelli Mundim, Ademir Franco, José Luiz Cintra Junqueira, Monikelly do Carmo Chagas Nascimento

**Affiliations:** 1 Division of Oral Radiology, Faculdade São Leopoldo Mandic, Instituto de Pesquisas São Leopoldo Mandic, Campinas, São Paulo (SP), Brazil; 2 Department of Oral Radiology, Centro Integrado de Radiodontologia, Goiânia, Goiás, Brazil; 3 Department of Therapeutic Stomatology, Institute of Dentistry, Sechenov University, Moscow, Russia; 4 Division of Forensic Dentistry, Faculdade São Leopoldo Mandic, Campinas, Brazil; Kuwait University, Faculty of Dentistry, KUWAIT

## Abstract

Intraoral scans became part of the virtual planning in Dentistry. In the new scenario of digital workflows, dental clinics and laboratories had to establish an online communication that requires the compression, decompression, and transmission of 3D files. Knowledge about the effects of these procedures on the dimensional properties of the files is fundamental to ensure a more realistic virtual planning. The aim of this study was to assess the influence of 3D file compression, decompression, and online transmission on the dimensional properties of dental models from intraoral scanning. Intraoral scan files in.stl format of 50 patients were selected from the database of a dental radiology clinic, with 25 of these patients with mixed dentition and 25 with permanent dentition. The maxilla and mandible scans of each patient were included in the study, generating a total of 100 files. A folder with the 100 files was created and replicated six times with different labels (A, B, C, D, E, F), totaling a sample number of 600 files. Folder A was compressed by WinZip and then decompressed. Folder B went through the same process, but the step of compression and decompression by WinZip was repeated 10 times. The folders C, D, E, F were sent, respectively, through the platforms WeTransfer, Dropbox, Google Drive, and OneDrive, then each of them was downloaded in their respective platforms. After the six folders went through the compression process and were sent by the platforms, each file in the folder was compared with its original file by superimposing the 3D images and identifying the dimensional deviation in the compressed file in relation to the original file. We observed that there were no differences between the six groups regarding dimensional changes from the compression, decompression and online transmission processes. The lack of dimensional changes was observed for the sets of permanent and deciduous. teeth We concluded that it is possible to compress, decompress, and transfer.stl format files online without causing dimensional distortions in the 3D model.

## Introduction

Development of new digital technologies such as intraoral scanning have generated significant changes in the processes of diagnosis and treatment planning in various areas of dentistry [1]. The three-dimensional image formed from the scanner is precise and does not show color, texture, or other common attributes of the computer-aided design (CAD) model [2]. Its most used digital format is the standard tessellation language/standard triangle language (STL) [3].

Digital models can be shared in seconds through networks and cloud services to local and foreign dental clinics, dental laboratories, or imaging centers. This minimizes the time needed to obtain the plaster model and reduces the cost of transportation and delivery [4]. However, due to the size and number of digital images in radiological imaging departments, there is increasing interest in using image compression to reduce the overall data size [5].

“Reversible" or "lossless" compression preserves all original pixel information after compression and decompression [6]. Whereas “irreversible” or “lossy” compression provides greater compression, even though it does not preserve the pixel values upon decompression, and recovers values that may be suitable for a specific purpose. The best known examples for these algorithms are JPEG and JPEG 2000 (syn. J2K), which can be used with both methods [7]. The use of irreversible compression tools must be tuned to diagnostic performance, so that they can be used without producing noticeably visible differences ["no visual loss"] and/or without producing differences that affect diagnostic performance ["no diagnostic loss"] [3].

The virtual planning with the use of three-dimensional (3D) models obtained from intraoral scans have more predictable and optimized results in dental specialties. However, for virtual planning to be replicated in patients, these models need to be accurate and dimensionally stable, favoring the planning of implants, prostheses, orthodontic treatments, and smile planning [3].

There is lack of information in the literature about the influence of compression, decompression, and online transmission of 3D images obtained with intraoral scanners. Thus, this study aimed to evaluate the influence on dimensional changes of the compression process and online transmission of images in STL format obtained by an intraoral scanner.

## Materials and methods

### Sample selection

The Research Ethics Committee of Faculdade Sao Leopoldo Mandic approved this study under number 4.356.616. This study was based on a retrospective collection of data from an existing image database. For this reason, informed consent was not applicable. Instead, the images were blindly analyzed by the main researcher after anonymization of data by removing from the intraoral scanning images, any personal information that could lead to the identification of the patients. To this end, an alphanumeric code was established for each participant. Intraoral scan images of 50 individuals, from a database of a private radiology clinic, were selected: 25 individuals with mixed dentition and 25 with permanent dentition. The following inclusion criteria were established: images of subjects with both maxilla and mandible scanned; files in STL format; and only the most recent scan of the patient, in case of more than one scan. Edentulous patients, and those who had orthodontic appliance were excluded from this study. Considering the maxilla and mandible scans of each patient included in the study, a total of 100 files were generated.

### Image acquisition

The images were collected retrospectively from an existing database. Previously, all the images that fed the database were obtained for diagnostic purposes or treatment planning with the TRIOS 3^®^ intraoral scanner (3Shape Inc., Copenhagen, Denmark) operated by a single technician and following the manufacturer’s guidelines.

### Analysis of the images

These files were copied directly from the computer to a hard disk and then transferred to a computer where the research was conducted. The folder with the 100 files was copied six times and separated into six different folders (A, B, C, D, E, F), totaling a sample number of 600 files. Folder A was compressed using WinZip (WinZip Computing, Inc., Mansfield, United States of America) and then decompressed. Folder B underwent the same process, but the WinZip compression and decompression step was repeated 10 times. The files of folders C, D, E, F were compressed once and then the folders were sent online, respectively, through the platforms WeTransfer, Dropbox, Google Drive, and OneDrive. Then, the download of each of them was performed from their respective platforms.

A preliminary approach was set in this study to verify if the compressed/decompressed and transferred 3D models remained the same after processing. To this end, an MD5 checksum was accomplished with the online tool MD5_File_Checksum–software (https://emn178.github.io/online-tools/md5_checksum.html). This tool can assess images from their metadata and cross-check for the similarity of the 32-character hexadecimal number computed for online files. The analysis of MD5 checksum is considered an option to assess data integrity from non-intentional corruption of files. In this study, we simulated routine procedures performed by health care professional while handling digital imaging. Hence, assessing the files in groups A, B, C, D, E, and F with the MD5 checksum tool was a preliminary step to provide inferences on data integrity.

After the six folders went through compression/decompression and transferring with the setups in groups from A to F, each file in the folder was pair-wise compared to its original file to check for dimensional changes (assessment of structural changes after the preliminary checksum analysis). This comparison was performed by the GOM engineering software (2019 Hotfix 6, Ver. 125216, Build 2020-02-27). In the software, the 3D models were visualized pairwise within each group of interest set in the present study (considering the processes of compression/decompression and transmission) Once imported, the 3D models were brought together in a pre-alignment condition [8] that considers a least square relationship between them. With the models in a close spatial relationship, the alignment process was subsequently “refined” with the computation of an additional best-fit for superimposition. The comparison between 3D models is quantified as micrometers (μm) of the deviation (distances) detected pairwise. Any detected deviation would reflect dimensional changes between the models–eventually influenced by the compression/decompression or online transmission of files. The analysis of the images was performed by a single evaluator (a maxillofacial radiologist with over 10 years of experience in practice), who repeated the superimpositions after 30 days.

### Statistical analysis

Once collected, the data were organized and submitted to statistical treatment, using the SPSS software, version 24.0 (IBM Corp. Released 2016. IBM SPSS Statistics for Windows, Version 24.0. Armonk, NY: IBM Corp.). Descriptive statistics were used to present the data for each of the groups (folders A, B, C, D, E, F). The results are presented in microns.

## Results

The preliminary analysis of our study consisted of the MD5 checksum assessment of the 3D model files. The hash for our matrix files was not changed after compression/decompression and transferring (groups A, B, C, D, E, and F). Taking as an example the permanent dentition 3D model, the original hash was “*92b13e3765c6968b38edb74e3ec38539*”, which remained the same after a single decompression, 10x decompression, and transferring with We Transfer, Dropbox, Google Drive, and One Drive. This outcome pointed towards a maintenance of file integrity after processing. Next, eventual dimensional changes were assessed with GOM software package

Analyzing the difference in structural dimensions in the different groups according to the compression software used and the online transmission tool, we found no dimensional changes in all intraoral scan models of mixed dentition (Table 1). For permanent teeth, the absence of difference was also observed among all 25 models analyzed (Table 2).

**Table 1:** Values of difference in structural dimensions in the different groups according to compression software and online transmission tool used in the mixed dentition scanning models.

Patients	WinZip^a^	WinRAR^a^	WeTransfer^b^	GoogleDrive^b^	Dropbox^b^	OneDrive^b^
**1–25**	0,000000	0,000000	0,000000	0,000000	0,000000	0,000000

a: software used for compression and decompression

b: software used for the transmission of.stl files.

**Table 2:** Values of difference in structural dimensions in the different groups according to the compression software and online transmission tool used in the scanning models of permanent dentition.

Patients	WinZip^a^	WinRAR^a^	WeTransfer^b^	GoogleDrive^b^	Dropbox^b^	OneDrive^b^
**1–25**	0,000000	0,000000	0,000000	0,000000	0,000000	0,000000

a: software used for compression and decompression

b: software used for the transmission of.stl files.

**Fig 1:**
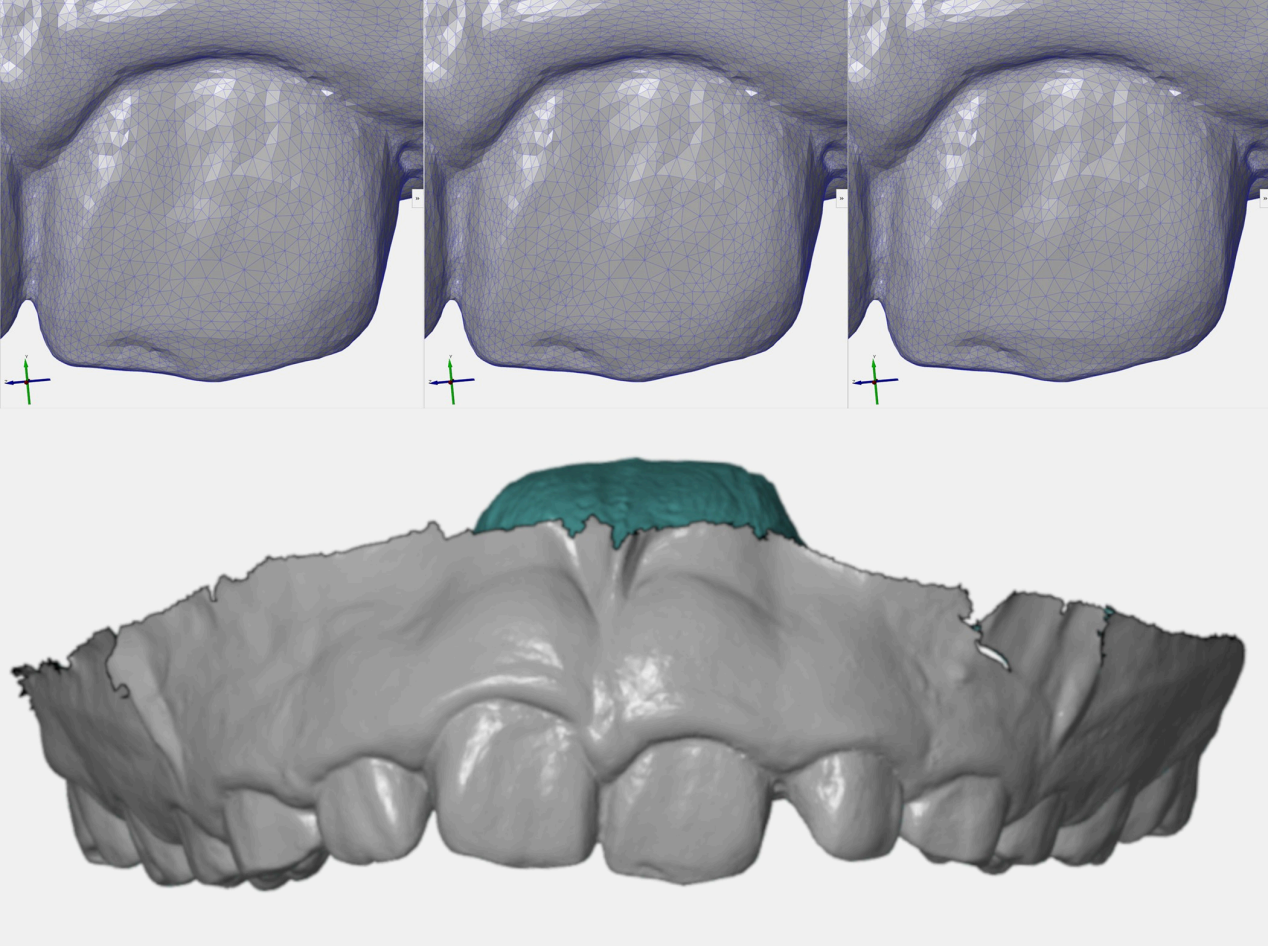
Comparative visualization of 3D models’ wireframe and superimposition for the permanent dentition. Original (A), decompressed/transferred (B), and superimposed (C) dental models in “zoom-in” visualization to enable the observation of object triangles (wireframe). An overview of the superimposed and pair-wise compared 3D dental models is provided (D) to show that no dimensional changes were detected in the permanent dentition.

**Fig 2:**
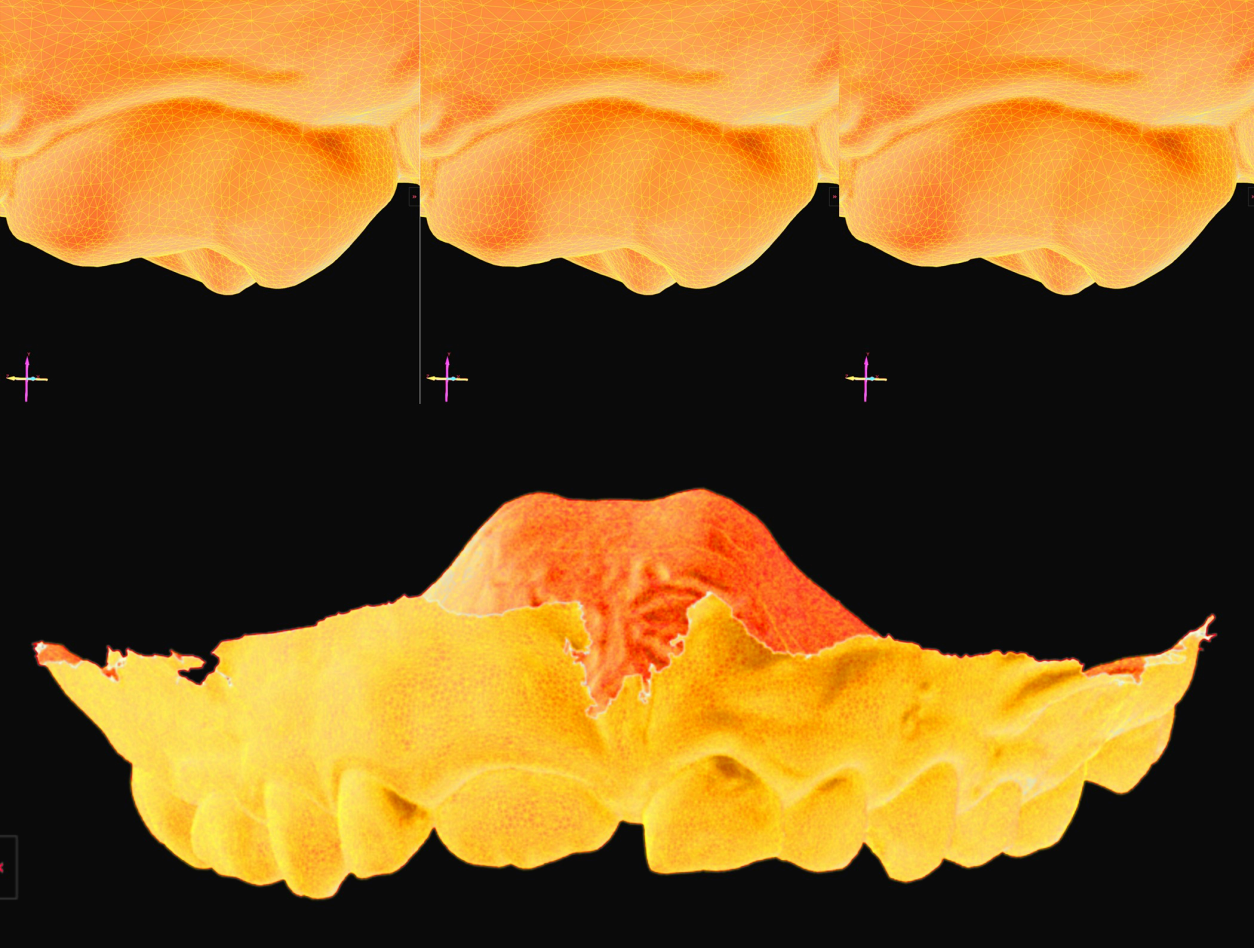
Comparative visualization of 3D models’ wireframe and superimposition for the deciduous dentition. Original (A), decompressed/transferred (B), and superimposed (C) dental models in “zoom-in” visualization to enable the observation of object triangles (wireframe). An overview of the superimposed and pair-wise compared 3D dental models is provided (D) to show that no dimensional changes were detected in the deciduous dentition. The images are presented with an inverted colour scheme and changes of colour balance to illustrate the 3D models from a different perspective of surface triangulation and superimposition.

Fig 1 shows a zoomed-in view of the original file of the permanent dentition, followed by the 3D model after decompression and by the superimposition of original and decompressed 3D models. Absence of structural deviation (d = 0.00μm) was observed for all the pair-wise comparison within the groups of interest in this study–namely files compressed/decompressed once (A), compressed/decompressed 10x (B), and files transferred via Dropbox (C), Google Drive (D), WeTransfer (E) and OneDrive (F). The same outcomes (d = 0.00μm) were observed for the 3D models of the deciduous dentition (Fig 2).

## Discussion

The continuous development in computer and data processing technology ensures new opportunities in the field of dentistry. The digitalization trend is a ubiquitous phenomenon currently in the dental community. The number of occurrences for the nonspecific search term "digital dentistry" in PubMed has more than doubled compared with results from ten years ago [9]. Among the topics of interest of dental clinicians and researchers, image data storage and transmission have fundamental importance given the increase of digital workflows in Dentistry. This study aimed to test the effects of compression, decompression, and online transmission on the accuracy of 3D models in STL format obtained via intraoral scanning.

Despite the numerous advances of scanners applied to dentistry, the STL format files generated by them present large volumes of data. This characteristic may be a limitation in its use, since it generates the need for a large amount of free space for storage in computers, servers, and difficulties in the process of online transmission [4]. To favor this process, data compression can be considered as a strategy to reduce storage space and improve data transfer rates [10, 11]. Thus, this study aimed to evaluate the influence of compression and online transmission of images in STL format obtained by intraoral scanners.

File compression is a process of intelligently restructuring data: instead of listing the same information repeatedly, compression lists only one and then creates a reference to the first one, every time that information appears in the file. Because this reference is smaller than the initial occurrence, the compressed file ends up being much smaller than the original. To create these references, the file compressor software has an algorithm that analyzes the entire content of the file and looks for the most repeated occurrences. These patterns are then added to the "dictionary," which translates the references to the original terms. It is noteworthy that larger files have a higher compression coefficient, since there are more repetitions and absence of a significant increase in the size of the dictionary. A compacted file can be identified by its.zip extension, the most commonly used format today [12].

Considering the results of this study, compression was used as a favorable tool for reducing the size of the STL files generated by intraoral scans. This factor is considered important when applied to the reality of radiological clinics or laboratories that perform the scanning and need to store the obtained files. This process also favors the professionals who will receive the file and store it to perform digital planning of patients, since they will need less digital space on their hardware. According to this study, regardless of the number of times that the compression process is performed [from one to 10 times], the final file will be similar to the original, without interfering in the diagnostic process.

Online data transmission apps have become an important tool in the area of health sciences, favoring the communication process between different professionals and even patients [13, 14]. These apps reduce geographical barriers, because they allow users to upload and store files, and to access them from anywhere, as long as they have internet access. The main highlight of the service is the possibility of freeing up space on local devices, such as computers and servers. Instead of consuming memory to store files locally, the user can store them on the platform, freeing up local space for other features. Another important fact of the apps is the automatic backup of the saved files, which avoids loss of dental records of patients by the dentist or radiology clinic, corroborating with the compliance with the ethical duty of keeping the clinical data that constitute the patient’s medical record [15, 16].

As for the variable of application for online data transmission, no difference was observed among the tools Dropbox, Google Drive, WeTransfer, and OneDrive, which is extremely satisfactory for clinical application, since all these developers present possibilities of free use of the tools. Other studies have evaluated these online transmission tools and their influence on the diagnosis of dental conditions such as caries and fractures. The results show no influence of these apps on the quality of the diagnosis [17, 18].

The association of the compression process verified by this study and the subsequent online transmission of STL files is a feasible tool in the radiological clinical routine and applicable, without loss of relevant information, considering the methodology proposed by this study. In this study, STL files of intraoral scans of children and adults were included and assessed with software and online tools commonly used to handle digital files. Further studies that seek to evaluate other compression software as well as new online transmission apps are encouraged.

## Conclusion

Based on the data evaluated, we concluded that it is possible to compress and decompress and perform online transfer of STL files without producing dimensional distortions in the 3D model.
